# Alpha‐globin gene triplication and its effect in beta‐thalassemia carrier, sickle cell trait, and healthy individual

**DOI:** 10.1002/jha2.262

**Published:** 2021-07-19

**Authors:** Mohammad Hamid, Bijan keikhaei, Hamid Galehdari, Alihossein Saberi, Alireza Sedaghat, Gholamreza Shariati, Marziye Mohammadi‐Anaei

**Affiliations:** ^1^ Department of Molecular Medicine, Biotechnology Research Center Pasteur Institute of Iran Tehran Iran; ^2^ Research Center for Thalassemia and Hemoglobinopathy, Health Institute Ahvaz Jundishapur University of Medical Sciences Ahvaz Iran; ^3^ Department of Genetics, Faculty of Sciences Shahid Chamran University of Ahvaz Ahvaz Iran; ^4^ Department of Medical Genetics, Faculty of Medicine Ahvaz Jundishapur University of Medical Sciences Ahvaz Iran; ^5^ Department of Endocrinology Ahvaz Jundishapur University of Medical Sciences Ahvaz Iran; ^6^ Narges Medical Genetics and PND Laboratory Ahvaz Iran

**Keywords:** alpha‐globin triplication, beta‐thalassemia, Iran

## Abstract

The genotype and phenotype correlation between coinheritance of heterozygous beta‐thalassemia with the alpha‐globin triplication is unclear. In this study we have investigated and reviewed alpha triplication frequency in beta‐thalassemia carriers, sickle cell trait, and healthy individuals and its effect on hematological and phenotypical changes. In this study, 4005 beta‐thalassemia carriers, 455 sickle cell trait, and 2000 healthy individuals were included. Molecular characterization of beta and alpha‐thalassemia was performed. The frequencies of alpha‐globin triplication in beta‐thalassemia carriers, sickle cell trait, and healthy individuals were 67 (1.67%), 4 (0.88%), and 18 (0.9%), respectively. In total, the frequency of alpha‐triplications is approximately 89 (1.39%) in Khuzestan province, South of Iran population. We have compared the average hematological parameters of beta‐thalassemia carriers, sickle cell trait, and healthy individuals with and without alpha gene triplication.

This mutation did not show any significant effect on the change of blood indices, neither in healthy individuals nor in sickle cell trait and beta‐thalassemia carriers. Therefore, there is no need to take more notice of anti 3.7 mutation in beta‐thalassemia carriers is opposed with some studies reported that the presence of excess alpha‐globin genes in beta‐thalassemia carriers can lead to the phenotype of beta‐thalassemia intermedia. Therefore, not every individual with triplicated alpha globin coinherited with beta‐thalassemia trait will have a significantly lower Hb than normal, and it is highly likely that none of them will need transfusion.

## 1 INTRODUCTION

Between the major, transfusion‐dependent forms of the disease and the symptomless carrier states is a thalassemia intermedia which is a milder form of the disease needing fewer or no transfusion and consequently less or no iron chelation. Generally, the level of hemoglobin in those who are affected by intermediate thalassemia is below 9–10 g/dl [1]. Beta‐thalassemia is further classified into severe (hemoglobin level as low as 4–5 g/dl, transfusion‐dependent, clinical symptoms similar to β‐thalassemia major), moderate (hemoglobin levels between 6 and 7 g/dl, transfusion‐independent, clinical symptoms similar to β‐thalassemia intermedia), and mild (hemoglobin levels between 9 and 12 g/dl, transfusion‐independent, usually do not develop clinically significant problems) clinical forms [2]. Alpha triplication is, in fact, an increase in alpha genes that occurs on one of the chromosomes. Alpha triplication mechanism is an unequal crossover during the recombination of α1 and α2 hemoglobin, which is the establishment of ααα^anti3.7^ triplicated allele. The clinical and hematological picture of beta‐ thalassemia heterozygotes with a triplicate α‐globin gene arrangement is variable, ranging from an asymptomatic presentation to a mild to moderate thalassemia intermedia phenotype [3, 4, 5, 6]. This study aims to investigate the frequency of alpha triplication mutations in beta‐thalassemia carriers and healthy individuals, and its effects on the red blood cell indices in the south of Iran.

## 2 PATIENTS AND METHODS

### 2.1 Ethical statement

This study was approved by the Ethics Review Committee of Pasteur Institute of Iran. Informed consent was signed and obtained from all participants following a detailed description of the purpose of the study. All methods were carried out in accordance with relevant guidelines and regulations.

### 2.2 Study subjects

The peripheral blood was taken from those referred to Narges Genetics Laboratory in Ahvaz city including 4005 beta‐thalassemia carriers, 455 sickle cell trait, and 2000 healthy individuals from Khuzestan province, south of Iran. Hematological indices were automatically measured on a Coulter Counter ABX Micros 60 (Helena Laboratories, Beaumont, TX, USA). Hemoglobin electrophoresis was performed on cellulose acetate (Helena Laboratories) using Tris‐EDTABorate buffer (pH 8.4). The Khuzestan Province is located southwest of Iran with a population of about 4.7 million people based on the 2016 census with different ethnicities (Arab, Lur, Bakhtiaris, and Fars). In this study 1773 beta‐thalassemia carriers and 55 sickle cell trait with different mutations of alpha thalassemia were excluded. So, we just selected beta‐thalassemia carriers, sickle cell trait, and healthy individuals with the normal genotype of alpha thalassemia to compare with those who had alpha‐triplications

### 2.3 Molecular studies

Molecular studies were conducted on genomic DNA isolated from peripheral blood cells by a salting‐out procedure [7]. For identifying *α*‐thalassemia genotype, investigation of common Mediterranean *α*‐globin gene deletions (‐α^3.7^, ‐α^4.2^ ‐α^20.5^ and –^MED^) and ααα^anti 3.7^ triplication were performed [8]; the entire *α‐* and *β‐*globin genes were amplified and DNA sequenced, ABI ‐3130 (Applied Biosystems, Foster City, CA, USA) [9]. In some cases, multiplex ligation‐dependent probe amplification (MLPA assay) was performed using the SALSA MLPA kit P140‐B4 HBA (MRC‐Holland, Amsterdam, Netherlands).

### 2.4 Data analysis

The results were examined using *t*‐test, two tails and were compared on the *p*‐value < 0.05 significance level.

## 3 RESULTS

### 3.1 Frequency of alpha‐globin gene triplication

In this study, 4005 beta‐thalassemia carriers, 455 sickle cell trait, and 2000 healthy individuals were chosen. The frequencies of alpha‐globin gene triplication in three studied groups including beta‐thalassemia carriers, sickle cell trait, and healthy individuals were 67 (1.67%), 4 (0.88%), and 18 (0.9%) respectively. So, it could be said that about 0.9% of the normal population, 1.67% of the beta‐thalassemia carriers, and 0.88% sickle cell trait have anti 3.7 mutation in the south of Iran with different ethnicities (Arab, Lur, and Fars). In total, 89 alpha‐triplication mutations have been detected among 6460 individuals tested. Therefore, in total the frequency of *α*‐triplications is approximately 1.39% in Khuzestan province, south of Iran population.

### 3.2 Hematological parameters

The average hematological parameters of every studied genotype with alpha gene triplication in three groups were compared with the same mutations with the normal of the alpha‐globin gene. Alpha gene triplication did not show any significant role in the changing of blood indices, in healthy individuals, sickle cell trait, and beta‐thalassemia carriers. All beta‐thalassemia heterozygotes with triplicated *α*‐globin genes, were clinically asymptomatic, and none of them needed a blood transfusion. The data are summarized in Table 1. In this study the level of total hemoglobin in thalassemia intermedia is considered between 6 and 9 g/dl; hence, most of the beta‐thalassemia carriers with 19 different mutations associated with alpha triplication were higher than 9 g/dl of hemoglobin. We have just had four beta‐thalassemia carriers with alpha triplication (4 of 66, 6%) lower than 9 g/dl of hemoglobin (one female CD36/37‐T/wt, 7.9 g/dl, one male IVSII‐I (G‐A)/ wt, 8.6 g/dl, one female CD82‐83(‐G)/ wt, 8.5 g/dl, and one female CD8(‐AA)/ wt, 8.6 g/dl) which none of them received blood transfusion.

**TABLE 1:** Comparing of average hematological parameters in carrier of beta thalassemia, sickle cell trait, and healthy individuals with and without alpha triplication

*β* genotype	*α* genotype	*n*	Gender female/male	Age mean ± SD	MCV (fL) mean ± SD	MCH (pg) mean ± SD	Hb (g/dl) mean ± SD	RBC (10^12^/L) mean ± SD	Hb A (%) mean ± SD	Hb A2 (%) Mean ± SD	Hb F (%) Mean ± SD
wt/wt	ααα^anti 3.7^/αα	18	8/10	26 ± 4.72	81.47 ± 5.76	26.05 ± 1.26	13.5 ± 1.2	5.13 ± 0.46	96.97 ± 0.46	2.58 ± 0.27	0.5 ± 0.23
*p*‐value					0.79	0.75	0.88	0.83	0.63	0.76	0.9
CD36/37‐T/wt	αα/αα	265	137/128	26.7 ± 5.2	62.15 ± 3.36	19.03 ± 1.2	11.36 ± 1.3	6.08 ± 0.90	93.73 ± 1.24	5.25 ± 0.87	0.84 ± 0.73
CD36/37‐T/wt	ααα^anti 3.7^/αα	17	9/8	27 ± 9.4	59.64 ± 4.8	18.45 ± 1.03	10.88 ± 1.21	5.6 ± 0.74	94.55 ± 0.40	5.07 ± 0.77	0.47 ± 0.22
*p*‐value					0.022	0.10	0.29	0.072	0.208	0.54	0.33
IVSII‐I(G‐A)/ wt	αα/αα	239	135/104	25.65 ± 7.0	63.32 ± 3.56	19.52 ± 1.36	11.37 ± 1.46	5.84 ± 0.82	94.02 ± 1.30	4.71 ± 0.95	1.46 ± 1.02
IVSII‐I(G‐A)/ wt	ααα^anti 3.7^/αα	10	5/5	27.7 ± 4.41	61.05 ± 2.01	19.58 ± 1.48	10.98 ± 1.56	5.54 ± 0.75	93.77 ± 3.05	4.97 ± 0.37	0.9
*p*‐value					0.088	0.91	0.52	0.35	0.78	0.59	?
CD44(‐C)/ wt	αα/αα	112	50/62	24.7 ± 3.2	62.79 ± 2.85	19.17 ± 0.79	11.46 ± 1.42	6.00 ± 0.66	94.38 ± 0.62	4.92 ± 0.53	0.57 ± 0.23
CD44(‐C)/ wt	ααα^anti 3.7^/αα	5	2/3	30.3 ± 3.3	58.50 ± 0.71	18.00 ± 2.82	10.40 ± 0.14	5.90 ± 0.28	94.45 ± 0.21	5.40 ± 0.28	?
*p*‐value					0.062	0.192	0.328	0.835	0.894	0.265	?
5′UTR+20(C‐T)/ wt	αα/αα	160	52/108	27.1 ± 3.35	61.79 ± 3.10	18.67 ± 0.87	12.05 ± 0.91	6.46 ± 0.57	94.15 ± 0.77	5.26 ± 0.71	0.52 ± 0.25
5′UTR+20(C‐T)/ wt	ααα^anti 3.7^/αα	5	2/3	24.0 ± 5.5	66.50 ± 0.28	18.40 ± 0.56	11.3 ± 0.141	6.03 ± 0.05	93.45 ± 0.64	5.15 ± 0.21	?
*p*‐value					0.066	0.687	0.292	0.335	0.294	0.845	?
CD82‐83(−G)/ wt	αα/αα	123	62/61	29.0 ± 8.1	62.92 ± 2.9	19.66 ± 2.15	11.29 ± 1.52	5.78 ± 1.04	93.74 ± 1.16	5.00 ± 0.66	1.12 ± 0.82
CD82‐83(−G)/ wt	ααα^anti 3.7^/αα	4	2/2	26.0 ± 8.5	61.00 ± 0.14	18.95 ± 0.63	11.7 ± 1.70	?	?	?	?
*p*‐value					0.382	0.650	0.724	?	?	?	?
CD8(‐AA)/ wt	αα/αα	175	120/55	29.4 ± 8.8	64.06 ± 2.3	19.87 ± 0.95	10.40 ± 0.95	5.37 ± 0.59	93.73 ± 0.59	4.98 ± 0.77	1.14 ± 0.66
CD8(‐AA)/ wt	ααα^anti 3.7^/αα	3	3/0	22.3 ± 5.1	62.45 ± 0.77	20.80 ± 0.28	10.2 ± 1.13	4.68 ± 0.17	94.5 ± 0.70	4.85 ± 0.820	?
*p*‐value					0.359	0.207	0.780	0.132	0.150	0.820	
−28(A‐C)/ wt	αα/αα	125	62/63	25.4 ± 8.7	70.73 ± 3.02	22.33 ± 1.18	12.72 ± 1.08	5.71 ± 0.4	94.27 ± 0.57	4.91 ± 0.76	0.67 ± 0.32
−28(A‐C)/ wt	ααα^anti 3.7^/αα	2	1/1	23 ± 0.7	72.85 ± 0.63	22.7 ± 1.41	12.50 ± 0.70	4.8 ± 0.28	?	?	?
*p*‐value					0.338	0.679	0.776	0.844			
IVSI‐6(T‐C)/ wt	αα/αα	126	59/67	27.14 ± 6.8	70.77 ± 4.71	21.83 ± 1.79	12.68 ± 1.55	5.81 ± 0.55	95.99 ± 0.73	3.58 ± 0.74	0.61 ± 0.31
IVSI‐6(T‐C)/ wt	ααα^anti 3.7^/αα	2	1/1	25.5 ± 2.1	72.20 ± 0.28	22.6 ± 0.42	12.0 ± 0.3	5.24 ± 0.19	95.30 ± 0.14	3.85 ± 0.07	0.60 ± 0.14
*p*‐value					0.676	0.558	0.638	0.158	0.206	0.62	0.978
Fr8‐9(+G)/ wt	αα/αα	127	61/66	26.0 ± 5.2	62.82 ± 2.98	19.13 ± 1.33	11.36 ± 1.28	5.97 ± 0.69	94.09 ± 0.96	5.13 ± 0.86	0.8 ± 0.53
Fr8‐9(+G)/ wt	ααα^anti 3.7^/αα	3	1/2	23.5 ± 2.1	59.0 ± 1.41	18.5 ± 0.71	10.25 ± 1.06	5.50 ± 0.71	94.0 ± 0.42	5.5 ± 0.42	0.5 ± 0.0
*p*‐value					0.088	0.512	0.249	0.355	0.90	0.569	0.461
CD15(TGG‐GA)/ wt	αα/αα	125	60/65	26 ± 5.7	62.67 ± 2.30	19.25 ± 0.95	12.07 ± 1.31	6.33 ± 0.73	94.03 ± 0.68	5.05 ± 0.97	0.62 ± 0.42
CD15(TGG‐GA)/ wt	ααα^anti 3.7^/αα	2	1/1	24 ± 1.41	60.75 ± 4.45	18.43 ± 1.03	10.9 ± 1.55	5.90 ± 0.51	94.25 ± 1.48	4.95 ± 0.92	0.45 ± 0.07
*p*‐value					0.291	0.255	0.245	0.422	0.724	0.890	0.579
Indian deletion/ wt	αα/αα	10	5/5	29 ± 7.7	58.94 ± 2.60	18.72 ± 1.23	10.97 ± 2.17	5.867 ± 1.09	96.53 ± 1.34	2.92 ± 0.64	0.66 ± 0.47
Indian deletion/ wt	ααα^anti 3.7^/αα	3	3/0	25 ± 2	59.56 ± 2.97	17.9 ± 0.17	12.15 ± 0.21	5.39 ± 0.11	94.9 ± 0.14	3.3 ± 0.14	1.7 ± 0141
*p*‐value					0.727	0.290	0.480	0.486	0.20	0.467	0.064
−101(C > T)/ wt	αα/αα	64	34/30	26 ± 5.4	79.48 ± 4.5	26.2 ± 1.9	14.0 ± 1.76	5.36 ± 0.64	95.82 ± 0.9	3.4 ± 0.5	0.86 ± 0.62
−101(C > T)/ wt	ααα^anti 3.7^/αα	3	1/2	24 ± 2.0	77.2 ± 0.32	26.3 ± 1.2	15.6 ± 0.97	5.93 ± 0.2	95.7 ± 0.5	3.5 ± 0.25	0.5 ± 0.2
*p*‐value					0.45	0.92	0.14	0.15	0.9	0.74	0.58
IVSI‐110(G > A)/ wt	αα/αα	180	93/87	27.2 ± 6.5	64.8 ± 5.3	20.6 ± 1.7	12.01 ± 1.28	5.91 ± 0.65	94.5 ± 1.9	4.5 ± 0.6	0.96 ± 0.81
IVSI‐110(G > A)/ wt	ααα^anti 3.7^/αα	3	2/1	25.6 ± 3.2	66.9 ± 10.9	21.7 ± 4.9	12.1 ± 2.4	5.6 ± 0.25	93.3 ± 2.7	4.3 ± 1.02	2.3 ± 1.7
*p*‐value					0.52	0.33	0.94	0.41	0.31	0.67	0.019
IVSI‐I(G > A)/ wt	αα/αα	111	60/51	28.4 ± 5.2	63.02 ± 3.73	19.21 ± 1.37	11.47 ± 1.20	5.63 ± 0.73	94.38 ± 2.90	4.46 ± 0.9	1.28 ± 0.99
IVSI‐I(G > A)/ wt	ααα^anti 3.7^/αα	1	1/0	?	63	19.4	10.6	4.6	?	?	?
*p*‐value					?	?	?	?	?	?	?
CD5(‐CT)/ wt	αα/αα	43	21/19	26.1 ± 4.7	63.29 ± 3.80	19.42 ± 1.09	11.59 ± 1.30	6.03 ± 0.7	94.58 ± 0.88	5.03 ± 0.71	0.76 ± 0.60
CD5(‐CT)/ wt	ααα^anti 3.7^/αα	1	1/0	28	63.3	18.6	10.2	5.7	?	?	?
*p*‐value					?	?	?	?	?	?	?
IVSI‐5(G > C)/ wt	αα/αα	144	78/66	27 ± 5.4	65.2 ± 4.7	20.1 ± 1.9	11.9 ± 1.4	5.8 ± 0.6	94.9 ± 0.7	4.2 ± 0.6	0.76 ± 0.5
IVSI‐5(G > C)/ wt	ααα^anti 3.7^/αα	1	0/1	26	67	21.5	9.0	5.71	?	?	?
*p*‐value					?	?	?	?	?	?	?
Initiation CD(T > C)/wt	αα/αα	40	16/24	26 ± 3.1	73.74 ± 8.8	23.07 ± 3.3	12.72 ± 1.29	5.56 ± 0.7	96.35 ± 1.46	3.15 ± 1.3	0.5 ± 0.4
Initiation CD(T > C)/wt	ααα^anti 3.7^/αα	1	0/1	26	54.6	17.4	12.6	7.25	?	5.8	?
*p*‐value					?	?	?	?	?	?	?
CD39 (C > T)/ wt	αα/αα	63	32/31	28.4 ± 6.8	62.7 ± 6.74	19.65 ± 1.3	11.42 ± 1.28	5.85 ± 0.69	94.4 ± 1.26	4.63 ± 1.2	0.86 ± 0.72
CD39 (C > T)/ wt	ααα^anti 3.7^/αα	1	0/1	22	61.6	20.1	11.5	5.73	93.3	6.1	0.6
*p*‐value					?	?	?	?	?	?	?
Hb S/ wt	αα/αα	400	220/180	25.85 ± 4.5	83.73 ± 4.32	28.22 ± 1.97	13.78 ± 1.71	4.86 ± 0.56	57.11 ± 4.83	2.65 ± 0.81	0.61 ± 0.53
Hb S/ wt	ααα^anti 3.7^/αα	4	1/3	24.3 ± 10.0	85.95 ± 6.5	27.5 ± 1.34	13.96 ± 0.86	5.17 ± 0.11	56.72 ± 2.27	3.12 ± 0.38	0.27 ± 0.38
*p*‐value					0.355	0.47	0.854	0.280	0.874	0.261	0.276

## 4 DISCUSSION

There are different studies which showed the frequency of the alpha‐globin gene triplication in healthy individuals and thalassemia patients. The frequency of the alpha‐globin gene triplication is varied, and it is dependent on the prevalence of thalassemia disease and some selection mechanisms such as endemic malaria in the studied countries [3, 10].

The highest carrier frequency of the anti 3.7 mutation was observed in 4.0% in healthy individuals of Indian, and lowest carrier frequency was reported in Malay population with 0.59%. In this study from the 2000 healthy individuals, 18 had alpha triplication mutation, it can be stated that the frequency of this allele in healthy people in Khuzestan province, particularly in Ahwaz city, equals 0.9% which is lower than previous Iranian study and more than the other countries (Table 2). The following table shows the prevalence of this triplication in the healthy individuals in Iran and other populations of the world.

**TABLE 2:** Allele and genotype frequency of ααα^anti3.7^ in normal subjects in this study and other populations

Population	Number of chromosomes studied	Allele frequency of ααα^anti3.7^	Genotype frequency of ααα^anti3.7^ (%)	References
Normal subjects				
Mexican	84	0.01	2.4	[20]
Togolese	342	0.011	2.3	[20]
Kenyan	114	0.008	1.6	[20]
South African blacks	306	0.01	1.96	[20]
Cypriote	990	0.01	2	[20]
Namibian	202	0.01	1.98	[20]
Portuguese	200	0.020	4	[20]
Indian	1856	0.004	0.75	[21]
Indian	536	0.011	2.2	[22]
Indian	2550	0.02	4	[23]
North Indian	416	0.017	3.36	[24]
Chinese	500	0.008	1.6	[20]
Southern Chinese	2338	0.0047	0.94	[25]
Chinese	1020	0.0098	1.96	[22]
Malay	1014	0.003	0.59	[22]
Thai	430	0.007	1.39	[26]
North Morocco	3316	0.0006	0.12	[27]
Iranian	794	0.01	2	[28]
Our study	4000	0.0045	0.9	

Our results also showed that the frequency of the alpha‐globin gene triplication in the Khuzestan population including hemoglobinopathies and normal individuals is 1.39%; this result is close to the results obtained from our country in previous studies. The lowest carrier frequency of alpha‐globin gene triplication including hemoglobinopathies and normal individuals was observed in Omani with 0.47%, whereas the higher frequencies were in other populations, including 10% in Mexican, Saudi Arabian 3.9% and 3.1% in North Indian population (Table 3).

**TABLE 3:** Total frequency of ααα^anti3.7^ in this study and other populations including hemoglobinopathies and normal individuals

Population	Individuals studied	Genotype frequency of ααα^anti3.7^ (%)	References
Turkish	225	5 (2.2%)	[33]
Saudi Arabian	104	4 (3.9%)	[34]
Omani	634	3 (0.47%)	[35]
North Indian	419	13 (3.1)	[24]
Indian	1253	15 (1.1)	[21]
Dutch	3500	42(1.2%)	[10]
Mexican	109	11 (10%)	[20]
Iranian	4010	69 (1.7%)	[14]
Iranian	1700	20 (1.2%)	[19]
Our study	6404	84 (1.31%)	

In this study, we also investigated the average hematological parameters of every studied genotype with alpha gene triplication in comparison with the same mutations with the normal of the alpha‐globin gene as well. We did not find any significant role in the changing of blood indices, only a marginal difference in beta‐thalassemia carriers if any (Table 1). We have just had four beta‐ thalassemia carriers with alpha triplication lower than 9 g/dl of hemoglobin (one female CD36/37‐T/wt, 7.9 g/dl, one male IVSII‐I (G‐A)/ wt, 8.6 g/dl, one female CD82‐83(‐G)/ wt, 8.5 g/dl, one female CD8(‐AA)/ wt, 8.6 g/dl); none of them received blood transfusion.

The general idea about the additional *α* gene is that the extra *α* gene aggravates the mild phenotype of the β‐thal carrier to the thalassemia intermedia of mild severity, but in most studies, the genotype and phenotype correlation between coinheritance of heterozygous β‐thal and the *α*‐globin triplication remains unclear [11, 12, 13, 14].

Previous studies showed that HbA2 and fetal hemoglobin levels were increased and very significantly reduced Hb level in the presence of *α*‐globin gene triplications in association with β‐thalassemia [12, 15, 16]. We have not observed a significant role in phenotype and hematological indices in individuals who carried different heterozygous *β*‐globin gene mutations with the *α*‐globin triplication.

Although we expected that extra chains of *α* globin gene eliminated by proteolysis did not have a significant effect or very limited effect in phenotype and hematological parameters.

In this study, our result was similar to some previous reports [10, 15, 17, 18]. For instance, Giordano et al found that none of the 12 *β*‐thal carriers with six different mutations and in association with an *α*‐triplication had a history of blood transfusion. Xiong et al also described 74 individuals co‐inheritance *β* thalassemia in carrier status and *α*‐triplication all presented the phenotype of the *β*‐thal trait. Our observations are precisely in keeping with the result that the presence of a triplicated *α*‐globin allele in *ß*‐thalassemia heterozygotes is associated with a phenotype of *β*‐thalassemia minor. This information is of importance in terms of genetic counseling.

In this study, we also evaluated previous data reported similar genotypes of our studies from patients with triplicated *α*‐globin genes and heterozygous *β*‐thalassemia for Hb (g/dl), transfusion‐dependent, and splenectomy. Of 37 affected persons who coinheritance of the *β*‐thalassemia heterozygotes (IVSII‐I (G‐A)/ wt) with triplicated alpha‐globin genes, 18 (48.6%) were transfusion‐dependent, four underwent splenectomy, and four had Hb less than 9 g/dl. In comparison with previously reported mutations, the results of previous Iranian studies [14, 19] reported from the same centers were different significantly. If we exclude these studies [14, 19], the results are changed fundamentally, where no one of individuals coinheritance of the β‐thalassemia heterozygotes (IVSII‐I (G‐A)/ wt) with triplicated alpha‐globin genes was transfusion‐dependent, no one underwent splenectomy, and just one had Hb less than 9 g/dl. It is suggested that the results of these two articles [14, 19] necessary to be re‐evaluated by a hematologist‐oncologist and verification of hematology analyzers (automated blood cell counters) are required.

In addition, of 59 *β*‐thalassemia heterozygote (CD39 (C > T)/ wt) with triplicated alpha‐globin genes, 11 (18.64%) were transfusion‐dependent, five (8.5%) underwent splenectomy, and 13 (22.0%) had Hb less than 9 g/dl. Due to comparing reported mutations in the previous studies, it can be recommended that the mutation of codon 39 needs to be taken into consideration and can have a clinical effect (Table 4).

**TABLE 4:** Previous studies reported similar genotypes of our studies from patients with triplicated *α*‐globin genes and heterozygous *β*‐thalassemia

					Transfusion‐dependent				
Population	β‐genotype	Number of patients	Hb (g/dl) Mean ± SD	Hb Less than 9 (g/dl)	No. of patients	Regular	Irregular	Splenectomy	References
Iranian	IVSII‐I(G‐A)/ wt	18	9.65 ± 0.2	?	14	6	8	2	[14]
Iranian	IVSII‐I(G‐A)/ wt	5	8.56 ± 0.7	3	4	2	2	2	[19]
Italian	IVSII‐I(G‐A)/ wt	3	11 ± 1.12	0	0	0	0	0	[29]
Brazilian	IVSII‐I(G‐A)/ wt	2	10.2 ± 1.6	0	0	0	0	0	[11]
Our study	IVSII‐I(G‐A)/ wt	9	10.65 ± 1.4	1	0	0	0	0	
Total	IVSII‐I(G‐A)/ wt	37	9.94 ± 1.43	4	18	8	10	4	
Total excluding [14, 19]	IVSII‐I(G‐A)/ wt	14	10.76 ± 1.26	1	0	0	0	0	
Iranian	CD15(TGG‐TGA)/ wt	1	8.2	1	1	1	0	1	[14]
Our study	CD15(TGG‐TGA)/ wt	2	10.9 ± 1.55	0	0	0	0	0	
Total	CD15(TGG‐TGA)/ wt	3	9.55 ± 1.9	1	1	1	0	1	
Greece	CD39 (C > T)/ wt	4	10.4 ± 1.1	0	0	0	0	0	[15]
European ancestry	CD39 (C > T)/ wt	5	9.1 ± 0.84	2	2	1	1	0	[30]
?	CD39 (C > T)/ wt	9	9.2 ± 1.6	4	4	0	4	2	[3]
Italian	CD39 (C > T)/ wt	18	9.93 ± 1.01	4	3	0	3	3	[29]
Italian	CD39 (C > T)/ wt	1	8.6	1	0	0	0	0	[31]
Dutch	CD39 (C > T)/ wt	15	10.1	?	0	0	0	0	[10]
Greece	CD39 (C > T)/ wt	6	9.86 ± 1.67	2	2	0	2	?	[36]
Our study	CD39 (C > T)/ wt	1	11.5	0	0	0	0	0	
Total	CD39 (C > T)/ wt	59	9.31 ± 1.22	13	11	1	10	5	
?	IVSI‐110(G > A)/ wt	3	10.1 ± 2.3	1	0	0	0	0	[3]
Italian	IVSI‐110(G > A)/ wt	1	8.9	1	0	0	0	1	[29]
Gypsy	IVSI‐110(G > A)/ wt	1	8.5	1	0	0	0	0	[32]
Dutch	IVSI‐110(G > A)/ wt	10	11.2	?	0	0	0	0	[10]
Greece	IVSI‐110(G > A)/ wt	2	8.7 ± 0.28	2	0	0	0	0	[36]
Our study	IVSI‐110(G > A)/ wt	1	10.2	0	0	0	0	0	
Total	IVSI‐110(G > A)/ wt	18	9.6 ± 1.0	5	0	0	0	0	
Iranian	IVSI‐5(G > C)/ wt	3	7.9	3	2	1	1	1	[14]
Iranian	IVSI‐5(G > C)/ wt	2	8 ± 0.42	2	2	1	1	1	[19]
Iranian	IVSI‐5(G > C)/ wt	2	12.1 ± 1.7	0	0	0	0	0	[28]
?	IVSI‐5(G > C)/ wt	2	10.8 ± 0.5	0	0	0	0	0	[3]
Our study	IVSI‐5(G > C)/ wt	1	9.0	0	0	0	0	0	
Total	IVSI‐5(G > C)/ wt	10	9.56 ± 1.83	5	4	2	2	2	
Total excluding [14, 19]	IVSI‐5(G > C)/ wt	5	10.63 ± 1.55	0	0	0	0	0	
?	IVSI‐I(G > A)/ wt	2	9 ± 1.4	1	0	0	0	1	[3]
Greece	IVSI‐I(G > A)/ wt	8	9.2 ± 1.3	3	0	0	0	0	[15]
Greece	IVSI‐I(G > A)/ wt	7	9.1 ± 1.8	4	4	0	4	?	[36]
Iranian	IVSI‐I(G > A)/ wt	2	10.1	?	2	1	1	1	[14]
Italian	IVSI‐I(G > A)/ wt	6	10.58 ± 1.2	?	0	0	0	1	[29]
Our study	IVSI‐I(G > A)/ wt	1	10.6	0	0	0	0	0	
Total	IVSI‐I(G > A)/ wt	26	9.76 ± 1.0	8	6	1	5	3	
Total excluding [14]	IVSI‐I(G > A)/ wt	24	9.70 ± 0.9	8	4	0	4	2	
Iranian	CD44(‐C)/ wt	1	9.6	0	0	0	0	0	[14]
Iranian	CD44(‐C)/ wt	1	9.8	0	0	0	0	0	[19]
Our study	CD44(‐C)/ wt	5	10.4 ± 0.1	0	0	0	0	0	
Iranian	CD8(‐AA)/ wt	2	9.4 ± 3.7	1	1	1	0	1	[14]
Iranian	CD8(‐AA)/ wt	1	7.8	1	1	1	0	0	[19]
Our study	CD8(‐AA)/ wt	3	10.2 ± 1.1	0	0	0	0	0	
Iranian	CD36/37‐T/wt	3	8.5 ± 1.83	2	2	1	1	0	[14]
Iranian	CD36/37‐T/wt	1	10.1	0	0	0	0	0	[19]
Our study	CD36/37‐T/wt	15	10.5 ± 1.6	1	0	0	0	0	
Iranian	Indian deletion/wt	1	7.3	1	1	1	0	0	[14]
Our study	Indian deletion/ wt	3	12. ± 0.2	0	0	0	0	0	
Dutch	CD5(‐CT)/ wt	11	8.7	?	0	0	0	0	[10]
Our study	CD5(‐CT)/ wt	1	10.2	0	0	0	0	0	
Dutch	IVSI‐6(T‐C)/ wt	15	14	0	0	0	0	0	[10]
Our study	IVSI‐6(T‐C)/ wt	2	12. ± 0.3	0	0	0	0	0	
Total		218	9.9 ± 1.2	42	45	17	28	17	
Total excluding [14, 19]		177	10.3 ± 1.0	28	15	1	14	7	

Finally, we evaluated all reported mutations in this and previous studies, for Hb (g/dl), transfusion‐dependent, and splenectomy. Of 218 *β*‐thalassemia heterozygote with triplicated alpha‐globin genes, 45 (20.64%) were transfusion‐dependent, 17 (7.8%) underwent splenectomy, and 42 (19.26%) had Hb less than 9 g/dl.

If we omit these studies [14, 19], the results are altered. The total numbers of studies persons are reduced to 177. Of 177 affected persons, 15 (8.47%) were transfusion‐dependent, seven (3.95%) underwent splenectomy, and 28 (15.82%) had Hb less than 9 g/dl where most of them coinheritance of the *β*‐thalassemia heterozygotes (CD39 (C > T)/ wt) with triplicated alpha‐globin genes. The data are summarized in Table 4.

In conclusion, the genotype of triplicated *α*‐globin gene and heterozygosity for *β*‐thalassemia mutation is not necessary to be considered as a cause of *β*‐thalassemia intermedia in our locality. Therefore, it is not essential to offer a prenatal diagnosis test to families and couples carrying an *α*‐globin gene triplication and a heterozygosity for *β*‐thalassemia. Therefore, not every individual with triplicated alpha globin coinherited with beta‐thalassemia trait will have a significantly lower Hb than normal, and it is highly likely that none of them will need transfusion. Due to previous studies, it can be recommended that the mutation of codon 39 needs to be taken into consideration.

## CONFLICT OF INTEREST

The authors declare that there is no conflict of interest that could be perceived as prejudicing the impartiality of the research reported.

## AUTHOR CONTRIBUTIONS

Mohammad Hamid directed the project, collected data, performed analysis, and wrote the manuscript. Bijan keikhaei, Alihossein Saberi, Gholamreza Shariati, Hamid Galehdari, Marziye Mohammadi‐Anaei provided the samples and clinical data. All of the authors reviewed and gave the final approval for the paper.

## FUNDING INFORMATION

This study was supported by grant number 687 from the Pasteur Institute of Iran, Tehran, Iran.

## Data Availability

All data generated during and/or analyzed during the current study are available upon request by contact the corresponding author.
